# Anti-Diabetic Activity and Metabolic Changes Induced by *Andrographis paniculata* Plant Extract in Obese Diabetic Rats

**DOI:** 10.3390/molecules21081026

**Published:** 2016-08-09

**Authors:** Muhammad Tayyab Akhtar, Mohamad Syakir Bin Mohd Sarib, Intan Safinar Ismail, Faridah Abas, Amin Ismail, Nordin Hj Lajis, Khozirah Shaari

**Affiliations:** 1 Laboratory of Natural Products, Institute of Bioscience, University Putra Malaysia, Serdang 43400, Malaysia; tayyabakhtar@hotmail.com (M.T.A.); syakir.sarib@gmail.com (M.S.B.M.S.); safinar@upm.edu.my (I.S.I.); faridah_abas@upm.edu.my (F.A.); nordinlajis@gmail.com (N.H.L.); 2 Faculty of Medicine and Health Sciences, University Putra Malaysia, Serdang 43400, Malaysia; aminis@upm.edu.my

**Keywords:** *Andrographis paniculata*, metabolomics, diabetes, obese-diabetic rat, type 2 DM, NMR

## Abstract

*Andrographis paniculata* is an annual herb and widely cultivated in Southeast Asian countries for its medicinal use. In recent investigations, *A. paniculata* was found to be effective against Type 1 diabetes mellitus (Type 1 DM). Here, we used a non-genetic out-bred Sprague-Dawley rat model to test the antidiabetic activity of *A. paniculata* against Type 2 diabetes mellitus (Type 2 DM). Proton Nuclear Magnetic Resonance (^1^H-NMR) spectroscopy in combination with multivariate data analyses was used to evaluate the *A. paniculata* and metformin induced metabolic effects on the obese and obese–diabetic (obdb) rat models. Compared to the normal rats, high levels of creatinine, lactate, and allantoin were found in the urine of obese rats, whereas, obese-diabetic rats were marked by high glucose, choline and taurine levels, and low lactate, formate, creatinine, citrate, 2-oxoglutarate, succinate, dimethylamine, acetoacetate, acetate, allantoin and hippurate levels. Treatment of *A. paniculata* leaf water extract was found to be quite effective in restoring the disturbed metabolic profile of obdb rats back towards normal conditions. Thisstudy shows the anti-diabetic potential of *A. paniculata* plant extract and strengthens the idea of using this plant against the diabetes. Further classical genetic methods and state of the art molecular techniques could provide insights into the molecular mechanisms involved in the pathogenesis of diabetes mellitus and anti-diabetic effects of *A. paniculata* water extract.

## 1. Introduction

Diabetes mellitus (DM) is a metabolic disease that occurs due to high blood glucose level, caused by insulin deficiency (Type 1 DM) or, quite often, combined with resistance (Type 2 DM) [1]. According to an International Diabetes Federation report (2014), 387 million people have been diagnosed with diabetes and that is expected to increase to 592 million by 2035. It has also been projected that Southeast Asia region will see the highest prevalence of DM around 2025 [2]. The World Health Organisation (WHO) estimates that 2.48 million people in Malaysia will have DM by the year 2030. A combination of factors are involved in the development or progression of diabetes e.g., genetics, environmental and nutritional effects. The disease, particularly Type 2 DM has turned into a global health issue and is becoming a major economic problem for the healthcare systems of many countries. It has become the third great ‘killer’ along with cancer and other metabolic diseases due to its high occurrence and mortality rate.

The scenario has significantly changed recently with the advent of the “back to nature” sentiments of consumers around the globe, triggered mainly by serious concerns over side effects associated with use of synthetics. Plants have been used as a traditional medicine for thousands of years and are proven to be a good source of new remedies against many life-threatening diseases. They are a significant source of drugs, which were derived directly or indirectly from them. Various examples have remarkably proved the innovative potential of natural compounds. Even the widely used antidiabetic drug metformin originated from the traditional medicine system of using *Galega officinalis* [3].

*Andrographis paniculata* (Burm.f) Nees (family Acanthaceae) is a popular medicinal plant in traditional Asian medicine and has been used in various traditional systems for centuries for the treatment of infectious diseases and associated fevers, as well as being a popular detoxifier, a carminative and tonic for liver and gastric problems [4]. Although native to India and Sri Lanka, it is widely cultivated in Southeast Asia including Malaysia. There have been numerous scientific reports on the pharmacological activities of *A. paniculata*, including its anti-inflammatory, anticancer, immunomodulation, anti-HIV, antibacterial, antispasmodic, antidiabetic, anticarcinogenic, antipyretic, hepatoprotective, anti-infective and antioxidant effects [5,6,7,8]. In China, the Traditional Chinese Medicine (TCM) formula, ‘Kan Jang’, is marketed as a herbal remedy for the common cold and respiratory problems. Among the many ethnomedicinal uses of *A. paniculata*, its antidiabetic property and its use as a remedy for liver dysfunctions figured prominently in Ayurveda and Chinese folk medicines.

Further to these reports, numerous labdane diterpenoids and flavonoids have been isolated from different parts of the plant. Andrographolide is the major diterpenoid while other similar diterpenoids such as deoxyandrographolide, neoandrographolide, 14-deoxy-11,12-didehydroandrographolide and isoandrographolide have also been isolated in varying quantities from the leaves of the species [9]. Several andrographolide analogues have also been synthesized and found to have potent anticancer [6,10,11] and glucosidase inhibitory activities [12].

Metabolomics is a qualitative and quantitative analysis of metabolites present in a biological system using a multi-analytical spectroscopy techniques along with multivariate statistical analysis [13]. Use of this approach to study the pharmacological effects of plants is fast gaining popularity due to its holistic nature and the information-rich results that can be obtained from it. The main applications of metabolomics are to identify biomarkers of disease progression and medical intervention, to investigate disease mechanisms and to provide additional information to support or aid the interpretation of genomic and proteomic data [14,15,16].

NMR spectroscopy is an unbiased and non-targeted approach and has been widely used to study the biofluids e.g., urine, plasma or serum samples for diagnostic and drug toxicity tests in human and animal based models [17]. NMR-based metabolomics facilitate the analysis of tissue or organ-specific toxicity and the identification of biomarkers associated with spcecific dieseases or toxicity [18]. A number of scientific reports have proved the analytical strength of NMR which can be utilized for the qualitative and quantitative analysis of a wide range of metabolites (biomarkers) present in the biofluids of diabetic rat models [19,20,21,22].

In the current study, *A. paniculata* was evaluated for its anti-diabetic effect on an obese-diabetic animal model. Despite various other reports or the anti-hypoglycemic activity of the plant, there has been no report describing metabolic alterations (or repairs) resulting from intervention with *A. paniculata* extract in the animal model. NMR-based metabolomics on animal body fluids are expected to shed light on the events that occur during the intervention and enable the identification of the specific biomarkers. This study is a crucial step in establishing the validity of the traditional claims of the efficacy of *A. paniculata* as a remedy for diabetes and set developing this herb into a phytomedicine.

## 2. Results

### 2.1. Amino Acid Profile of A. paniculata Leaf and Extract

**Figure 1:**
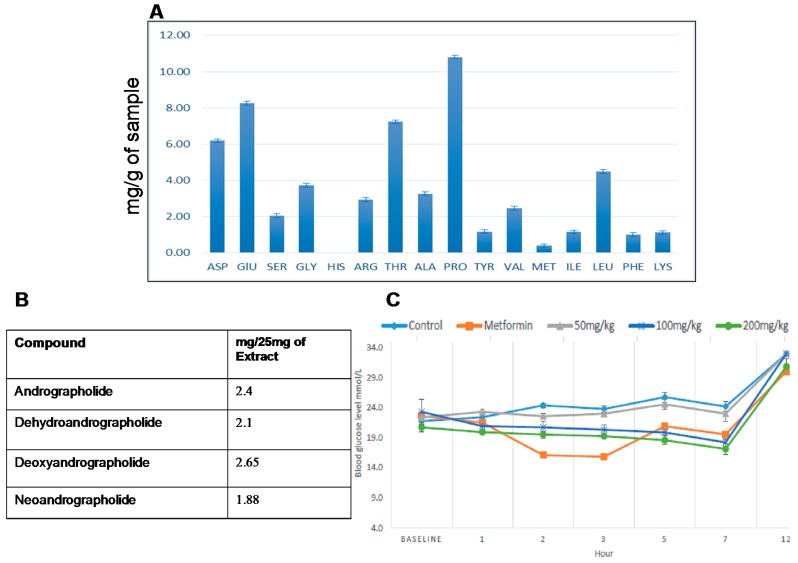
(**A**) Percentage content of individual amino acids of *A. paniculata* Harapan leaf water extract; (**B**) Quantitative ^1^H NMR analysis of four major chemical markers of *A. paniculata* leaf water; (**C**) Oral glucose tolerance test for obese–diabetic group treated and non-treated with *A. paniculata* harapan leaf water extract.

The total percentage of nitrogen in the *A. Paniculata* Harapan leaf was calculated to be 0.28%. The concentration of the individual amino acids identified in the leaf water extract (mg/g of sample) are given in Figure 1A. Proline, glutamine, threonine, asparagine and leucine were among the amino acids present in high quantities in the leaf water extract.

### 2.2. Quantitative Analysis of Selected Chemical Markers via qNMR

Quantitative analysis was carried out for the four chemical markers of *A. paniculata*. Diagnostic signals of andrographolide at δ 5.05 ppm, of dehydroandrographolide at δ 4.73 ppm, of deoxyandrographolide at δ 4.62 ppm, and of neoandrographolide at δ 4.56 ppm, were used for quantitative analysis relative signal of the IS at δ 5.53 ppm. The quantity of compound (mX) was measured by using the ratio of the integrals of the compounds and the (internal standard) IS, and the calculation was (where mIS is the content of IS; AX and AIS are the integrals; and MWIS and MWX are the molecular weights):

mX = (AX/AIS) × mIS/MWIS) × MWX

The quantitities of each of the four chemical markers on the basis of 25 mg of extract are given in Figure 1B.

### 2.3. Short–Term Toxicity Test on A. paniculata Leaf Water Extract

A short-term toxicity study of standardized extract of the *A. paniculata* Harapan leaf water extract was carried out on Sprague–Dawley rats. The animals were given 5, 50 and 300 mg/kg body weight water extract over a period of two weeks and the effect of the herb was monitored for any pharmacotoxic symptoms. There was no sign of toxicity and no deaths were recorded throughout the study. The effect of the extract on general behavior, BW, food and water intake, relative organ weight per 100 g BW, hematology, and clinical biochemistry was measured. There was no significant change found in any of the recorded parameters as compared to a control group which did not receive any extract. No gross pathological abnormalities were observed in the organs and the blood chemistry. Thus, the water extract of *A. paniculata* has no apparent short–term acute effect on the experimental animals at the given doses.

### 2.4. Oral Glucose Tolerance Test (OGTT) on A. paniculata Leaf Water Extract

Figure 1C showed that administration of 200 mg/kg of *A. paniculata* Harapan leaf water extract reduced glucose levels significantly, starting from the 1st hour and decreasing continuously until the 7th hour of the experiment. Groups treated with metformin, or 50 mg/kg or 100 mg/kg water extract of *A. paniculata* showed significantly reduced glucose levels starting from the 2nd hour, with the metformin group showing a drastic reduction of blood glucose. However, 50 mg/kg treatment showed no significant activity when compared to the control diabetic group. Non-fasting blood glucose levels recorded after 12 h showed that all groups reached HI value except for the groups treated with metformin or 200 mg/kg water extract.

### 2.5. Metabolite Analysis of Urine Samples

Metabolites in the urine samples collected from the experimental animal models were analyzed visually and via principal component analysis (PCA) to identify and quantify the metabolite changes associated with their physiological conditions.

**Figure 2:**
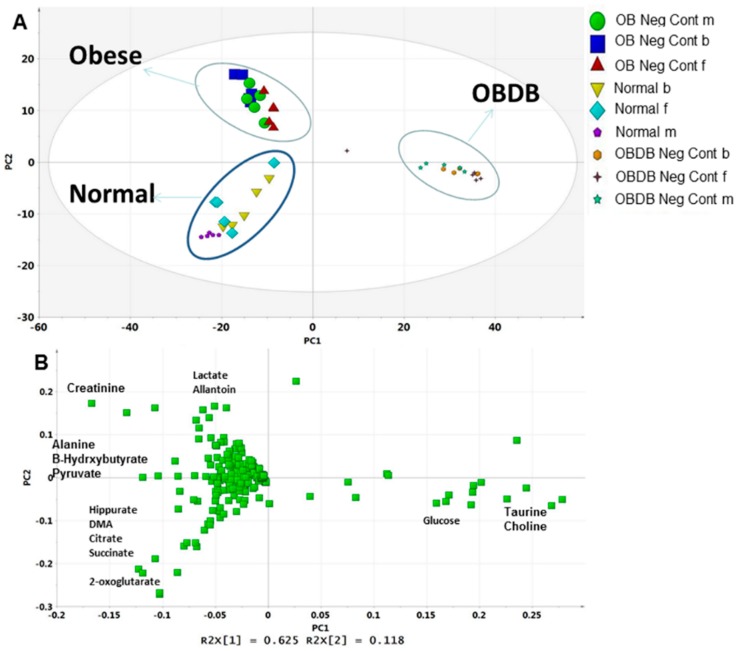
(**A**) PCA score plot of normal, obese, and obese–diabetic (obdb) rats; (**B**) PCA loading plot of normal, obese and obese-diabetic (obdb) rats.

Principal component analysis (PCA) was performed on the urine NMR data to identify the key metabolites regulated differently in normal, obese (ob), and obese–diabetic (obdb) rats. A clear separation of three groups with close grouping of replicates is shown in the PCA score plot (Figure 2A). Separation in the score plot suggested clear metabolic differences among normal, ob, and obdb rats. The score plot showed that 62% of separation is based on PC1 and 12% is based on PC2 score. PC1 was found to be responsible for the separation of obdb rats from normal and ob rats. Samples from ob and normal rats are separated by PC2, having positive and negative PC2 scores, respectively. The corresponding loading plot showed a higher level of glucose, choline, and taurine in obdb rats (Figure 2B), whereas, ob rats were marked by increased levels of lactate, creatinine, and allantoin. Higher quantities of hippurate, dimethylamine (DMA), citrate, succinate, and 2-oxoglutarate were found in normal rats. The relative quantification of all the metabolites in urine samples collected at the basal, middle, and final stages are shown in Supplementary Figures S6–S11.

**Figure 3:**
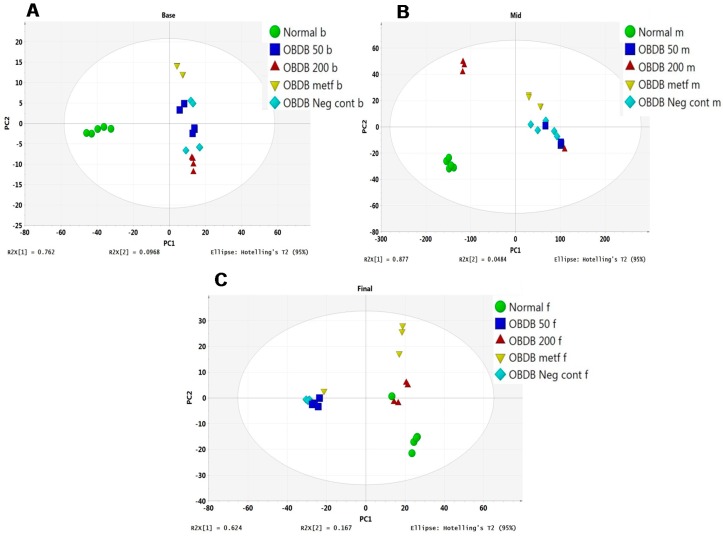
(**A**) PCA score plot of obese–diabetic and obese–diabetic treated rats’ urine samples collected at the basal stage. Normal (normal b), obese–diabetic (obdb neg cont b), obdb rats treated with 50 mg of *A. paniculata* extract (obdb 50 b), obdb rats treated with 200 mg of *A. paniculata* extract (obdb 200 b), and obdb rats treated with metformin (obdb metf b); (**B**) PCA score plot of obese–diabetic and obese–diabetic treated rats’ urine samples collected at the middle stage. Normal (normal m), obese–diabetic (obdb Neg cont m), obdb rats treated with 50 mg of *A. paniculata* extract (obdb 50 m), obdb rats treated with 200 mg of *A. paniculata* extract (obdb 200 m), and obdb rats treated with metformin (obdb metf m); (**C**) PCA score plot of obese–diabetic and obese–diabetic treated rats’ urine samples collected at the final stage. Normal (normal f), obese–diabetic (obdb Neg cont f), obdb rats treated with 50 mg of *A. paniculata* extract (obdb 50 f), obdb rats treated with 200 mg of *A. paniculata* extract (obdb 200 f), and obdb rats treated with metformin (obdb metf f). (“b” = basal stage, “m” = middle stage, “f” = final stage, “Neg cont” = Negative control).

Furthermore, PCA was applied to the urine NMR data of normal, obdb, non-treated (negative control), and obdb rats treated with metformin (positive control) or *A. paniculata* leaf water extract. At the basal urine collection, a clear separation was found among the normal, obdb negative control, and obdb treated groups in a PCA score plot (Figure 3A). Inspection of the score plot showed the clustering of a normal group at the negative side of PC1, whereas all other groups had positive PC1 scores. At the middle urine collection stage, obdb samples treated with 200 mg of *A. paniculata* extract had shifted closer to the normal group, having a negative PC1 score, while other groups were clustered at the positive side of PC1 in the score plot (Figure 3B). However, the 200 mg treated and normal groups were separated by PC2, having positive and negative PC2 scores, respectively. At the final urine collection, obdb samples treated with 200 mg of *A. paniculata* extract were clustered together with the normal group at the positive side of PC1, while the metformin-treated group was also present on the same side of PC1 but differed from the 200 mg treated and normal rats on the basis of PC2 score (Figure 3C). Meanwhile, the negative control and 50 mg treated groups were clustered together, having negative PC1 scores, and were well separated from the 200 mg, metformin, and normal groups.

**Figure 4:**
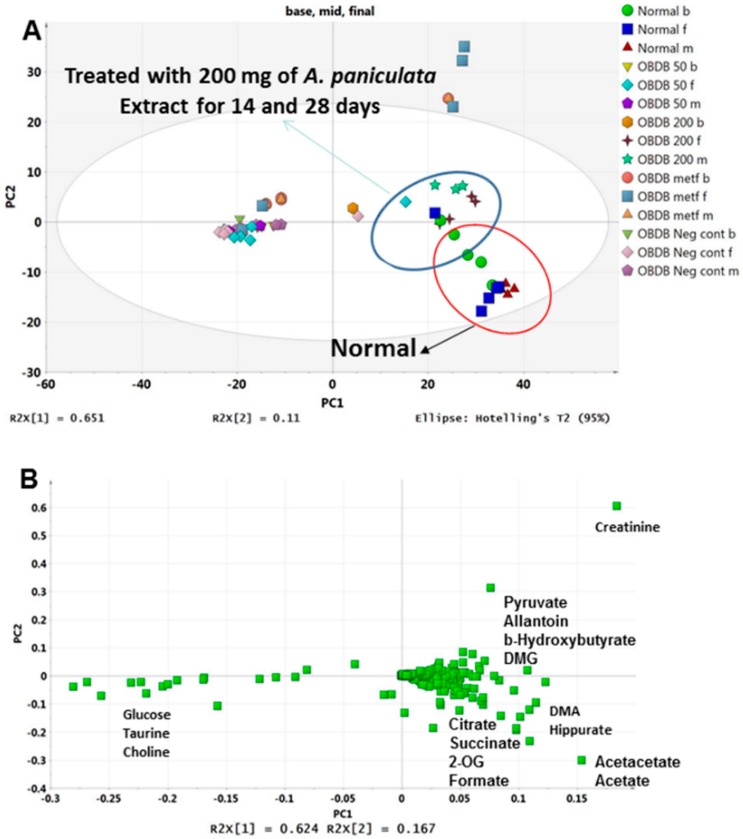
(**A**) PCA score plot of normal, obese–diabetic, and obese–diabetic treated (50 or 200 mg of *A. paniculata* extract, or metformin) rats’ urine samples collected at the basal, middle, and final stages; (**B**) PCA loading plot of normal, obese–diabetic, and obese–diabetic treated (50 or 200 mg of *A. paniculata* extract, or metformin) rats’ urine samples collected at the basal, middle, and final stages.

**Figure 5:**
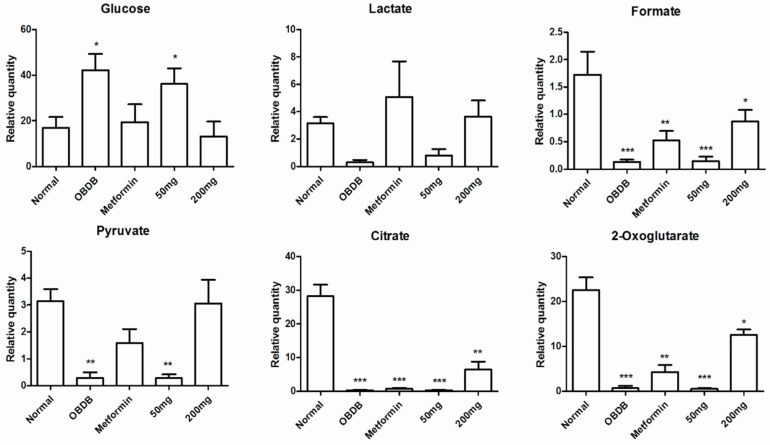

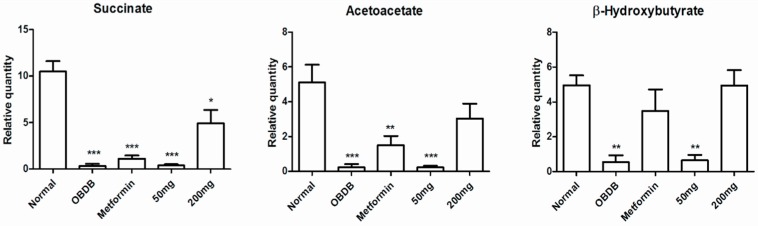
Relative quantification of the differentiating metabolites in urine samples (urine samples collected at final stage) of normal, obese–diabetic (obdb), and obese–diabetic rats treated with 50 or 200 mg of *A. paniculata* extract or with metformin. Relative quantification is based on mean peak area of the related ^1^H-NMR signals. * depicts the differences between normal (control), obese–diabetic (obdb), and obese-–diabetic rats treated with different concentrations of *A. paniculata* extract or metformin. Statistical icons: * *p* < 0.05, ** *p* < 0.01 and *** *p* < 0.001.

**Figure 6:**
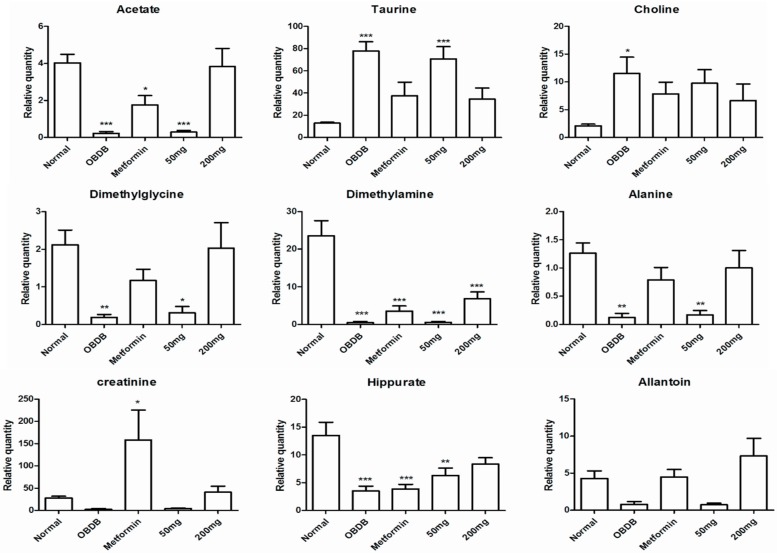
Relative quantification of the differentiating metabolites in urine samples (urine samples collected at the final stage) of normal, obese–diabetic (obdb), and obese–diabetic rats treated with 50 or 200 mg of *A. paniculata* extract or with metformin. Relative quantification is based on mean peak area of the related ^1^H NMR signals. * depicts the differences between normal (control) obese–diabetic (obdb), and obese–diabetic rats treated with different concentrations of *A. paniculata* extract or metformin. Statistical icons: * *p* < 0.05, ** *p* < 0.01 and *** *p* < 0.001.

These results indicated that 14 days of treatment with 200 mg water leaf extract of *A. paniculata* has a clear effect on the disturbed metabolome of obdb rats. Whereas continued exposure to the same dose (200 mg/kg BW) untill 28 days was able to partially restore the metabolic changes in obdb rats back towards the normal state. It can also be seen in the PCA score plot of pooled urine NMR data of all three stages (base, middle and final) that 14 and 28 days treated group with 200 mg *A. paniculata* extract are clustering close to normal group at the positive side of PC1 (Figure 4A). The corresponding loading plot (Figure 4B) showed higher levels of glucose, choline and taurine in the negative control and obdb group treated with 50 mg of *A. paniculata* extract. Whereas, higher quantities of citrate, succinate, 2-OG, formate, acetate, acetoacetate, DMA, hippurate, DMG, pyruvate and allantoin were associated with the normal and 200 mg treated groups. Meanwhile, at final urine collection, the PCA score plot showed the metformin treated group to be well separated from the normal and 200 mg treated groups. The metformin treated group was marked by high level of creatinine, as demonstrated by the loading plot. The relative quantification of all the metabolites regulated differently in normal, obdb and obdb treated group are shown in Figure 5 and Figure 6 (quantification of metabolites in urine samples collected at final stage) as well as in the Figures S1–S4 (quantification of metabolites in urine samples collected at basal and middle stages).

We also applied PCA to the urine NMR data of ob rats collected at the basal, middle, and final stages after treating with *A. paniculata* extract or metformin. We did not find any clear separation among the samples in the PCA score plot. Some uncorrelated variables, presence of noise, or systematic variations in NMR data might be the reason for poor separation in PCA. Orthogonal projection to latent structure analysis-discriminant analysis (OPLS-DA) was applied in order to remove this uncorrelated data. After applying OPLSD-DA, a clearer separation was found among the samples from normal, ob non-treated (negative control), and ob groups treated with *A. paniculata* or metformin (Supplementary Figure S5). PLS1 was found to be responsible for the separation of the normal group from the other groups. Neither of the ob groups treated with *A. paniculata* or metformin clustered close to the normal group. Rather, all the treated groups were projected closer to the non-treated ob group. These results indicated that *A. paniculata* did not exhibit anti-obesity activity in the obese rat model.

## 3. Discussion

Obesity is a fast-growing health issue and can lead to highly debilitating diseases such as diabetes, cardiovascular diseases and cancer [23]. It has been identified as one of the major syndromes correlated with diabetes, particularly in non-insulin–dependent diabetes (Type 2 diabetes) [23]. There have been many models developed to mimic human obesity and diabetic conditions. Here, we used a non-genetic out-bred Sprague-Dawley rat model fed with high-fat diet (HFD) for obesity and a combination of HFD along with a low-dose injection of streptozotocin (HFD + low STZ) to induce Type 2 diabetes-like conditions in rats. The latter model demonstrated many metabolic disturbances similar to human Type 2 diabetes and has also been used previously to identify in vivo biomarkers in rat urine samples related to diabetes [24,25].

Glucose metabolism is a major source of energy in most organisms. Glucose breaks into pyruvate via glycolysis. Under aerobic conditions, pyruvate is further converted into acetyl coenzyme A (AcCoA) via pyruvate dehydrogenase complex, which is the starting point of TCA cycle. However, under anaerobic conditions, instead of converting to AcCoA, pyruvate is converted into lactate through the lactate dehydrogenase enzyme. In our model of obesity we found higher levels of lactate, creatinine, and allantoin, whereas lower levels were observed for hippurate, dimethylamine, citrate, succinate, and 2-oxoglutarate. Previously, high concentrations of lactate have also been reported in the urine, blood, and tissue of obese mice and rats fed with HFD [26,27,28,29]. It was thus assumed that high lactate levels in obese animals are due to the upregulation of anaerobic glycolysis [26,28]. Moreover, subcutaneous fats are also known to be a rich source of lactate [30]. Therefore, higher lactate excretion in obese animals is also associated with larger adipose mass. These findings are also consistent with the higher lactate level in obese humans [31]. Conversely, a lower urine lactate level has been reported in the GK and STZ-induced diabetic rat models. This lactate reduction in the urine of STZ–administered rats was attributed to the lower production of ATP [32,33]. Depletion of ATP production has been linked with mitochondrial dysfunction in Type 2 diabetic conditions [28,34].

Citrate is also an important metabolite associated with insulin or glucose levels in metabolic studies. Citrate is a key metabolite in TCA cycle and converted into other metabolites (2-oxoglutarate, succinate, and fumarate) for energy production. In this study, lower levels of citrate along with 2-oxoglutarate and succinate were observed in the urine of obese rats compared to normal rats. Literature studies have reported lower urinary excretion of citrate in obese animals with insulin resistance [35], whereas an opposite effect has been seen in obese animals without insulin resistance [27]. The level of citrate urinary excretion is also dependent on the acid-base status of the body [36]. Alkalosis causes higher citrate urinary excretion while acidosis has the opposite effect. Obesity along with insulin resistance is known to induce metabolic acidosis, ultimately resulting in depletion of urinary citrate excretion [37]. These results are also in good agreement with data on human urine that showed lower citrate urinary levels in individuals suffering from insulin resistance [38].

In obese–diabetic rats, further reduction of citrate, 2-oxoglutarate and succinate, the intermediates of the TCA cycle, was also observed. These results indicated an alteration in energy metabolism after STZ injection in the obese rats. Our results are consistent with previously reported work on metabolic changes in STZ-induced diabetic rats [39]. Likewise, perturbation of energy related metabolites has been attributed to hyperglycemia and hypoinsulinemia after STZ treatment [39]. Published reports also demonstrated that alteration in energy metabolism can occur as a result of impairments in mitochondrial function [40], and inhibition of pyruvate kinase, glucokinase and phosphofructokinase in the presence of higher blood glucose levels [41,42].

Creatinine is a degraded product of creatine in muscles. It is biosynthesized from arginine and glycine. Creatinine production is directly proportional to body muscle mass, filtered through the kidney and excreted in urine [43]. Creatinine clearance rate is considered an indicator of renal function and stability. Many reports have demonstrated higher urinary excretion of creatinine in obese or HFD fed animals [44]. Hypertrophy of skeletal muscles is believed to be a major reason for higher urinary excretion of creatinine in obese animals [44]. Therefore, in order to regulate larger body mass and support kidney function, obese animals excrete more creatinine than lean or normal organisms [45]. Obesity is associated with insulin resistance, hypertension and other metabolic diseases that are potential risk factors for many kidney diseases and can lead to lower creatinine clearance with the passage of time [46,47]. In this study higher quantities of creatinine were found in the urine of obese rats compared to normal and obdb rats. In obdb rats, the creatinine level was low compared to normal rats. There could be multiple reasons behind the depletion of creatinine in diabetic rats, e.g., change in muscle mass, creatinine reabsorption , cell leakage, etc. These observations are also parallel to the findings showing lower creatinine clearance in Type 2 diabetic patients [20,48].

Taurine plays an important role in bile acid metabolism and has many important physiological roles such as antioxidation, neural modulation, and osmoregulation [49]. In our study, we found lower urinary taurine levels in obese rats compared to normal and obdb rats. Lower urinary and hepatic levels of taurine have also been reported in GHR mutant obese mice, along with lower expression of cysteine sulfinic acid dehydrogenase (Csad) [45]. Obese Zucker rats have also been reported to have lower taurine urinary excretion levels [34]. Lower urinary excretion of taurine has been attributed to lower hepatic taurine biosynthesis in mutant mice and this hepatic reduction is suggested due to inhibition of taurine biosynthetic enzymes which are closely linked to obesity [29,50]. Higher hepatic and lower urinary concentration of taurine along with lower expression of two key enzymes involved in taurine biosynthesis, cysteine dioxygenase (CDO) and Csad, have been reported in HFD-induced obese mice [51,52]. Oral supplementation of taurine has been reported to decrease the total weight of fat in the abdominal cavity and improve insulin sensitivity in a Type 2 diabetic rat model [50]. Contrary to what was observed for obese rats, higher taurine urinary excretion was found for obdb rats. These results are in agreement with previous reports that associated high urinary taurine excretion with poorly controlled diabetes in diabetic patients [53,54]. Another study demonstrated lower plasma and higher urinary excretion of taurine in Type 2 diabetic patients [55]. After an oral taurine load, the patients again had lower plasma and higher urinary concentration of taurine. These findings were linked to impaired renal reabsorption with enhanced urinary clearance and fractional excretion in diabetic patients. Elevated glucose level in diabetics have been said to disturb osmoregulation and can lead to osmotic stress in cells [49,56]. Since taurine is an osmolyte, it may be regulated differently in diabetic and normal organisms in order to maintain osmotic balance across the cell membrane.

Allantoin is the oxidative product of uric acid via purine catabolism [57]. Obesity is often combined with hyperuricemia. Uric acid is produced and secreted by adipose tissues through xanthine oxidoreductase (XOR). Obese mice were reported to have greater XOR activity and higher uric acid production compared to normal rats [58]. In this study higher allantoin urinary excretion was also found for obese rats compared to normal and obdb rats. This may be due to the higher quantity of adipose tissue mass in the obese animals, producing more urea which is further degraded into allantoin and excreted in the urine.

In the short-term toxicity test, no toxicity or significant adverse effects were observed in the rats treated with different doses of *A. paniculata* water extract. Even the highest dose (300 mg/kg body weight for two weeks) was found safe and did not incur mortality or changes in the general behavior of the treated animals. These results indicated that the oral use or administration of *A. paniculata* water extract is safe. In the oral glucose tolerance test (OGTT), a significant reduction was found in blood glucose levels of rats treated with metformin or the selected doses of *A. paniculata* extract, particularly the 200 mg/kg BW. These results indicated the anti-hyperglycemic activity of the plant extract and also provided us with a platform for using *A. paniculata* plant extract against Type 2 diabetes in the obese–diabetic rat model. 

In the metabolic study, 200 mg/kg BW of *A. paniculata* water extract partially restored the perturbed metabolites of obdb rats. At the basal stage, we found higher levels of glucose in obdb and obdb rats treated with metformin or 50 or 200 mg/kg BW of *A. paniculata* extract. After 14 and 28 days of treatment with metformin, a 200 mg dose of *A. paniculata* restored the glucose level back to normal, whereas no significant reduction of glucose was found in the case of a 50 mg dose of the plant extract. Pyruvate and lactate levels, which were lowered in obdb rats, regained normal levels after 28 days of treatment with metformin or 200 mg/kg BW of plant extract. The quantity of formate along with other TCA intermediaries (citrate, 2-oxoglutarate and succinate) was depleted in the urine of obdb rats. Treatment with metformin did not cause significant improvement in the obdb rats. In contrast, treatment with 200 mg/kg BW of plant extract caused a considerable increase in the quantity of formate and TCA intermediaries. However, the level of metabolites was not completely restored, even after 28 days of treatment, with the levels remaining significantly lower compared to normal rats.

Ketone bodies (acetate, acetoacetate, β-hydroxybutyrate), hippurate, and allantoin were also significantly reduced in obdb rats. The levels were completely restored after 14 and 28 days of treatment with 200 mg/kg BW of plant extract. Meanwhile, metformin could only restore the levels of β-hydroxybutyrate and allantoin, whereas the levels of acetate, acetoacetate, and hippurate remained significantly lower compared to normal rats, even after 28 days of treatment.

Apart from glucose, taurine and choline were the other metabolites found in higher concentrations in obdb rats. Treatment with metformin or 200 mg/kg BW of plant extract caused considerable reduction in the levels of both metabolites. After 28 days of treatment, there was no significant difference in the levels of metabolites compared to normal rats, although the quantitative graph trend showed higher quantities in all the treated groups. 

## 4. Materials and Methods

### 4.1. Chemicals and Reagents

Methanol-*d*_4_ (CD_3_OD, 99.8%), tetramethylsilyl propionic acid-*d*_4_ sodium salt (TSP), sodium deuterium oxide (NaOD), deuterated oxide (D_2_O, 99.9%), phosphate buffer (KH_2_PO_4_), streptozotocin (STZ), sodium azide (NaN_3_), metformin, and olistatin were purchased from Merck, Darmstadt, Germany. For the animal study, normal chow and high-fat diet (HFD) were purchased from Gold Coin, Kuala Lumpur, Malaysia.

### 4.2. Plant Material

#### 4.2.1. Large-Scale Planting *of A. paniculata* (Harapan) for Metabolomics Analysis of Biofluids

Seedlings of *A. paniculata* (Harapan) were obtained from Taman Pertanian Universiti (TPU), Universiti Putra Malaysia (UPM). The seedlings were acclimatized at the Institute’s medicinal plant nursery for a week before being transplanted (24 April 2012) to a small research plot belonging to the Biodiversity Unit of the Institute Bioscience, UPM. The agronomic care of the plant was according to the routine protocol of the unit where all plants were watered twice daily and fertilizer (NPK 15:30:15, Wellgrow, Selangor, Malaysia) was applied every once a week. Throughout the growth period no signs of pest attack or fungal disease were observed in the plants. The plants were harvested after 11 weeks (25 June 2012), i.e., just before the plants began flowering. Leaf and stem parts were separated while the plant materials were still fresh. Only the leaf material was utilized for the study.

#### 4.2.2. Processing of Plant Material for Animal Biofluids Study

*Andrographis paniculata* (Harapan) leaf material (45 kg) was oven-dried in a convection oven with temperature maintained at 40 °C. The drying process took about one week and the material was constantly monitored for fungal growth and other forms of degradation. About 11 kg of dried leaves were obtained and ground to a fine powder using a Wiley mill (0.70 mm mesh size).

#### 4.2.3. Preparation of *A. paniculata* Water Extract for Animal Study

The powdered leaf *A. paniculata* (Harapan) material was macerated in distilled water, at a plant:water ratio of 50:1. This was to simulate as close as possible the traditional use of the herb. The maceration process was assisted by sonication for 30 min, during which time the temperature of the water bath was maintained at 40 °C. The aqueous extract was filtered and concentrated down to a small volume using a pilot scale high vacuum rotary evaporator. During the concentration process the water bath temperature was maintained at 40–45 °C. The concentrated extract was frozen overnight in a freezer and then lyophilized to produce the final powdered extract, which was stored at −4 °C until needed.

### 4.3. Quantitative NMR Analysis of Selected Chemical Markers in A. paniculata (Harapan) Water Extract

Quantitation of the andrographolides was done according to [59]. ^1^H-NMR spectra of all the samples were recorded on a Varian 500 MHz NMR spectrometer (Varian Inc., Palo Alto, CA, USA), at 300 K, using the zg30 pulse sequence with 128 scans, spectra width of 5000 Hz, acquisition time of 3.2 s, and relaxation delay of 5.0 s. The spectral processing was done by using MestReNova (version 6.0.2-5475) (Mestrelab research S.L., A Coruna, Spain). Reference signals of IS at δ 5.53 ppm, of andrographolide at δ 5.05 ppm, of dehydroandrographolide at δ 4.73 ppm, of deoxyandrographolide at δ 4.62 ppm, and of neoandrographolide at δ 4.56 ppm were used for quantitative analysis. 

### 4.4. Determination of Total Nitrogen Content (Kjeldahl Method) in A. paniculata (Harapan)

In a micro Kjeldahl digestion flask, 0.15 g of powdered *A. paniculata* (Harapan) leaf sample was digested by boiling with 2.5 mL of concentrated H_2_SO_4_ and 0.8 g of mixed catalyst until the mixture was transparent and blue–green. After the temperature of the mixture reached 40 °C, 10 mL of distilled water were added and the digested product transferred to a distillation tube, fixed with a condenser. Fifty milliliters of 45% sodium hydroxide solution, 10 mL of 2% boric acid and a few drops of indicator were added to the digested product. Ammonia was steam distilled from the digested product, and excess acid in the flask was estimated by back titration against unreacted boric acid with 0.05 M sodium hydroxide solution until neutral. The nitrogen content (%) was calculated as follows:

$$\frac{\left(\mathrm{mL}\;\mathrm{standard}\;\mathrm{acid}\;-\;\mathrm{mL}\;\mathrm{blank}\right)\times N\;\mathrm{of}\;\mathrm{acid}\times 1.4007}{\mathrm{weight}\;\mathrm{of}\;\mathrm{sample}\;\mathrm{in}\;\mathrm{grams}}$$

where N is normality.

### 4.5. Amino Acid Analysis of A. paniculata (Harapan) Water Extract

The free amino acids present in the *A. paniculata* (Harapan) water extract were analyzed by an HPLC gradient system with a Merck C18 column. Buffer A (0.1 M ammonium acetate, pH 6.5) and buffer B (0.1 M ammonium acetate containing acetonitrile and methanol, 44:46:10, *v*/*v*, pH 6.5) were used.

For sample preparation, approximately 0.28 ± 0.10 g (14.29 mg protein) of *A. paniculata* (Harapan) leaf water extract was used and amino acid analysis was done as described by Hainida et al. [60].

### 4.6. Animals

Seventy-eight male Sprague–Dawley rats (around 150–250 g) were purchased from Saphire Enterprise Sdn Bhd, Selangor, Malaysia. The rats were placed in plastic cages with stainless steel covers. All the experimental work was conducted under the approval of Institutional Animal Care and Use Committee (IACUC), Faculty of Medicine and Health Science, Universiti Putra Malaysia (ACUC No: UPM/FPSK/PADS/BRUUH/00407). During the acclimatization period of one week (room temperature, i.e., 26–28 °C, 12/12 h light/dark cycle), the rats were given normal rat chow (Gold Coin, Malaysia) (14% fat, 61% carbohydrate, and 25% protein from total energy in kcal) and distilled water, ad libitum. After the acclimatization period the rats were randomly divided into three (3) groups of three rats (*n* = 3) for the toxicity study, three groups of six rats (*n* = 6) for the preliminary oral glucose tolerance test (OGTT), and two sets of groups comprising of control groups (normal, obese, and obese–diabetic) and treated groups that were treated with 50, 100, or 200 mg/kg BW *A. paniculata* (Harapan) leaf water extract or metformin as a positive control.

### 4.7. Toxicity Test

The toxicity test was performed to calculate an LD_50_ value and was carried out according to the OECD Guideline 423. Prior to the test, Sprague–Dawley rats were fasted overnight (12 h), after which they were weighed and divided into four groups of three (*n* = 3). Each animal was administered with the *A. paniculata* (Harapan) leaf water extract via gavage. Four different doses were used, i.e., 0, 5, 50, and 300 mg/kg body weight. The animals were not fed with any food for a further 3–4 h after treatment with the test extract. After treating with plant extract, each animal was observed individually once during the first 30 min; special attention was paid for the first four hours, and daily thereafter for 14 days. In case the animal was found dead or sacrificed for humane reasons, the time of death was noted as accurately as possible. Each animal was weighed shortly before treating with test substance, and weekly thereafter.

### 4.8. Oral Glucose Tolerance Test

A preliminary oral glucose tolerance test (OGTT) was conducted to evaluate the hypoglycemic properties of leaf water extract of *A. paniculata* (Harapan). A total of 30 Streptozotocin (35 mg/mL) induced obese–diabetic rats were divided into five groups of six. The first group served as a negative control (obese–diabetic) that received normal saline (0.9% NaCl) only; the second group received metformin (250 mg/kg); and the third, fourth, and fifth groups received 50 mg/kg, 100 mg/kg, and 200 mg/kg *A. paniculata* water extract, respectively. All the rats were made to fast overnight (12 h) prior to conducting the OGTT. All treatments were given orally using a G18 animal feeding needle with single dose and blood glucose measurement was taken by pricking the tail vain. Blood glucose levels were measured before *A. paniculata* extract treatment and at 1, 2, 3, 5, 7, and 12 h after glucose administration (2 g/kg BW). 

### 4.9. Animal Disease Models (Obese and Obese–Diabetic)

The obese animal model was developed according to Jalil et al. [61]. The animals were fed with a high-fat diet (HFD) for three months in order to make them obese. After three months of HFD, when they were deemed to have become obese (according to the Lee measurement), the obese rats were intravenously injected with STZ (25 mg/kg body weight) to induce diabetes. The plasma glucose was determined after three days of STZ injection (prepared in 0.05 M citrate buffer solution, pH 4.5) and the measurement was repeated on day 7 to confirm the symptoms of diabetes in rats. The literature shows that rats are considered diabetic when their basal plasma glucose level is higher than 13.9 mmol/L [62].

#### 4.9.1. Administration of *A. paniculata* Water Extract to Experimental Animals

Forty-eight normal male Sprague–Dawley (SD) rats (150–200 g) were housed in a room with controlled temperature (21 °C ± 2 °C ) and a 12 h light/dark cycle at Animal House, Kulliyah of Pharmacy, International Islamic University Malaysia (IIUM). Three doses (50, 100, and 200 mg/kg) of *A. paniculata* leaf water extract were prepared in 0.03% (*w*/*v*) of carboxymethylcellulose (CMC) and injected daily to the experimental animals by gastric intubation using a force-feeding needle. The animals were treated continuously for four weeks. During this interval, rats in the control groups (normal, obese, and obese–diabetic) received 0.03% (*w*/*v*) CMC. The metformin- (250 mg/kg) treated group was labeled as the positive control. The blood glucose level, food intake, and weight were recorded weekly starting from the baseline until 28 days of treatment. After data was recorded, urine collector fit on individual metabolic cages was used to collect from the control groups and treated groups, at the initial, baseline, middle, and final time points. The total volume of urine for each rat was recorded; all the urine samples were preserved with sodium azide (0.03%) and kept at –80 °C for further analysis. Obesity was assessed based on the Lee obesity index by dividing the cube root of the body weight (g) with the nose–anus length (cm) and multiplied by 1000. Values greater than 310 were considered obese. The animals were anesthetized with ketamine (75–100 mg/kg) before they killed. Blood was then obtained from each animal by cardiac puncture and stored in sample tubes prefilled with ethylenediaminetetraacetic acid (EDTA). Both serum and plasma were collected after centrifuging at 1000 RPM for 10 min at 4 °C.

#### 4.9.2. Extract Preparation and Dosage Calculation for Animal Study

The dried powder of AP extract was diluted with sterilized distilled water using the following formula.

$$\mathrm{Volume}\;\mathrm{to}\;\mathrm{be}\;\mathrm{given}\;(\mathrm{mL})=\frac{\left[(\frac{\mathrm{Body}\;\mathrm{weight}\;(g)}{1000})\times \mathrm{dose}\;(\mathrm{mg})\right]}{\left[\mathrm{stock}\;\mathrm{extract}]\;(\frac{\mathrm{mg}}{\mathrm{mL}}\right)}$$

The extract was freshly prepared before each experiment to prevent any possibility of degradation.

### 4.10. ^1^H-NMR Metabolomics Analysis of Biofluid Samples

After continuous feeding of a high fat diet for three months, urine samples from all the animal groups (control and treatment) were collected starting at week 12 (the initial time point). After inducing diabetes, a second urine collection was made at week 13 (the basal urine collection), a third urine collection was taken (after two weeks of treatment with *A. paniculata* water leaf extract or metformin) at week 15 (the middle urine collection), and, after further treatment for two weeks, a final urine collection was made at week 17 (final urine collection stage). Urine samples were collected in tubes, and 0.1% sodium azide was added as an anti-microbial agent. The collected urine samples were stored at –80°C prior to analysis. 

#### NMR Measurement and Data Processing

Nuclear magnetic resonance (500 MHz Varian NMR spectrometer (Varian Inc., Palo Alto, CA, USA) was used to identify the differentiating metabolites in the biofluids of metformin-treated, *A. paniculata*-extract-treated, and non-treated (control group) rats. For ^1^H-NMR analysis, urine samples were prepared and analyzed as described by Maulidiani et al. [63] The spectral processing of NMR data was done using Chenomx NMR Suite software (Version 7.1, Chenomx Inc., Edmonton, AL, Canada). The residual signals of water (δ_H_ 4.68–5.00), urea (δ_H_ 5.55–6.00), and imidazole (δ_H_ 7.23–7.40 and 8.43–8.46 ppm) were subtracted from the analysis. Spectral intensities were scaled to TSP and spectral region from δ_H_ 0.52–10.00 was binned into regions of 0.04 ppm width. Pareto scaling was applied to processed data and multivariate data analysis was performed using SIMCA-P+ version 12.0.1.0 (Umetrics AB, Umeå, Sweden). Statistical analysis was performed using GraphPad prism for Windows (version 5.03, Redmond, WA, USA). The NMR signals were assigned according to the existing literature databases (HMDB, http://www.hmdb.ca/; KEGG, http://www.genome.jp/kegg/; Chenomx NMR Suit Ver.7.1). Table 1 shows the identified metabolites in the urine of normal, obese, and obese–diabetic rats.

## 5. Conclusions

Our results showed differences in the metabolic profiles of obese, obdb, and normal rats that are consistent with previously published data. Some of the metabolites may be regulated or reported differently in other studies, which could be due to time-dependent alterations, different diets, age factors, or the use of different models. Treatment with 200 mg/kg BW of *A. paniculata* plant extract was found to be quite active in restoring the disturbed metabolic profile of obdb rats back to normal conditions. Further NMR-based metabolic studies along with classical genetic methods and state-of-the-art molecular techniques can lead to insight into the mechanisms involved in the development and progression of diabetes mellitus and the anti-diabetic effects of *A. paniculata* water extract.

## Figures and Tables

**Table 1:** Metabolites identified in normal, obese, and obese–diabetic rats.

Pathway	Metabolite	Chemical Shift (ppm)
Glucose metabolism	Glucose	4.64 (d, *J* = 8.0 Hz), 5.24 (d, *J* = 3.5 Hz)
	Pyruvate	2.32 (s)
	Lactate	1.32 (d, *J* = 7.5 Hz)
	Formate	8.40 (s)
TCA Cycle	Citrate	2.56 (d, *J* = 16.0 Hz), 2.68 (d, *J* = 16.0 Hz)
	Fumarate	6.52 (s)
	Succinate	2.40 (s)
	2-Oxoglutarate	3.00 (t, *J* = 7.0)
Fatty acid metabolism	β-Hydroxy butyrate	0.96 (t, *J* = 7.5 Hz)
	Acetoacetate	2.28 (s)
	Acetone	2.24 (s)
	Acetate	1.96 (s)
Choline metabolsim	Choline	3.20 (s)
	Dimethylglycine	2.92 (s)
Amino acid metabolism	Malonate	3.12 (s)
	Taurine	3.44 (t, *J* = 6.5 Hz); 3.27 (t, *J* = 6.5 Hz)
	Alanine	1.48 (d, *J* = 7.5 Hz)
	Hippurate	7.52 (t, *J* = 7.5 Hz); 3.98 (d, *J* = 7.5 Hz)
Creatine metabolism	Creatine	3.02 (s); 3.92 (s)
	Creatinine	4.04 (s), 3.04 (s)
	Urea	5.8 (br s)
TMA metabolsim	Dimethylamine	2.72 (s)
	Trimethylamine	2.88 (s)
Purine metabolsim	Allantoin	5.4 (s), 6.02 (s)

## Supplementary Materials

Supplementary materials can be accessed at: http://www.mdpi.com/1420-3049/21/8/1026/s1.
