# Supplementary material for: Anti-Diabetic Activity and Metabolic Changes Induced by Andrographis paniculata Plant Extract in Obese Diabetic Rats
Source: Molecules. 2016 Aug 9;21(8):1026. doi: 10.3390/molecules21081026 (PMC6273188; doi:10.3390/molecules21081026)
Supplement: Supplementary file 1 [file molecules-21-01026-s001.pdf]

Supplementary Materials: Anti-Diabetic Activity and Metabolic Changes Induced by *Andrographis paniculata* Plant Extract in Obese Diabetic Rats

Muhammad T. Akhtar, Mohamad S. Bin Mohd Sarib, Intan S. Ismail, Faridah Abas, Amin Ismail, Nordin Hj Lajis and Khozirah Shaari

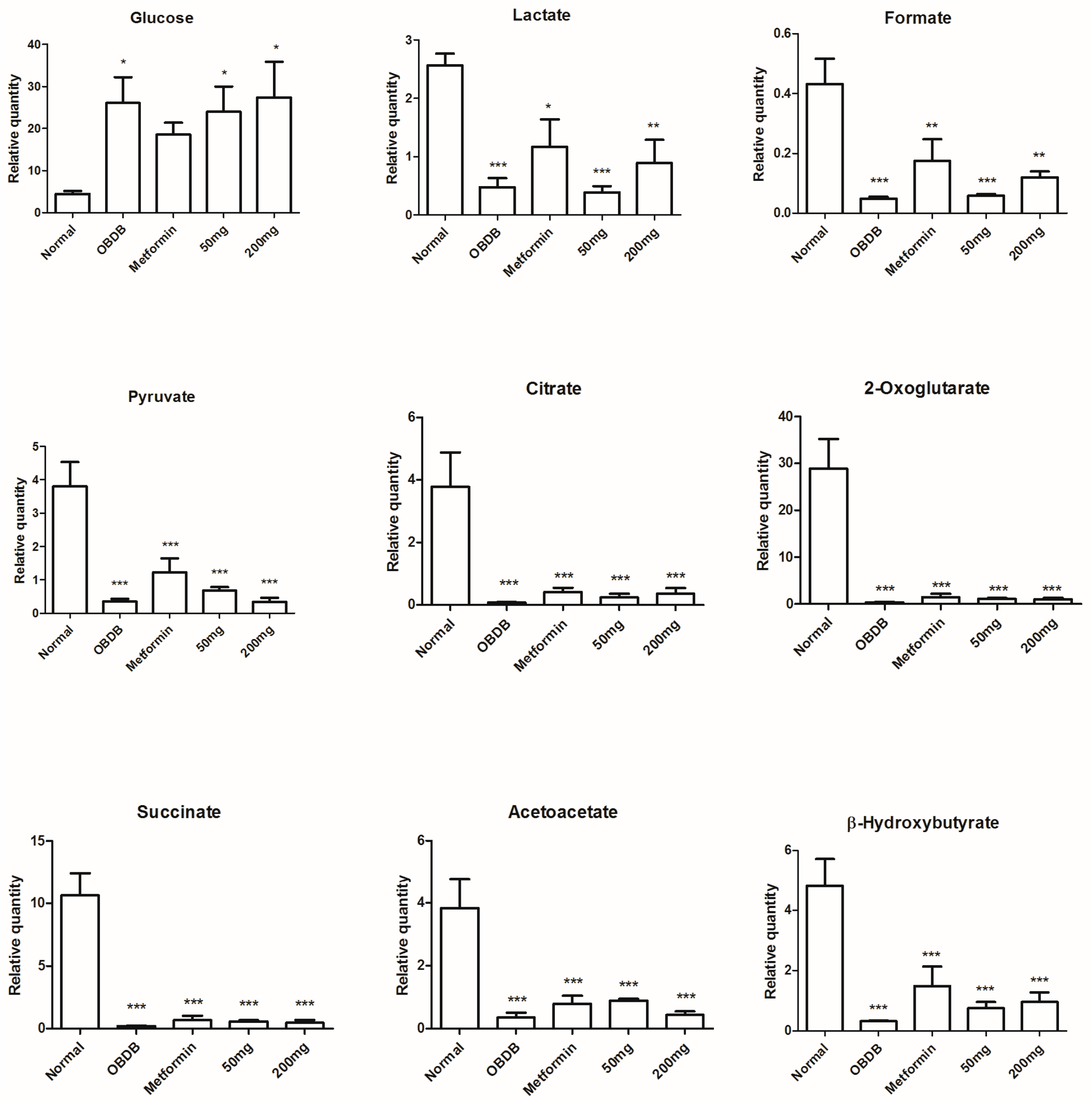

Figure S1. Relative quantification of the differentiating metabolites in urine samples (urine samples collected at basal stage) of normal, obese-diabetic (obdb), obese-diabetic rats treated with 50, 200 mg of *A. paniculata* extract and metformin. Relative quantification is based on mean peak area of the related ^{1}H NMR signals. * depict the differences between normal (control) and obese-diabetic (obdb), obese-diabetic rats treated with the different concentrations of *A. paniculata* extract and metformin. Statistical icons: * $p < 0.05$, ** $p < 0.01$ and *** $p < 0.001$.

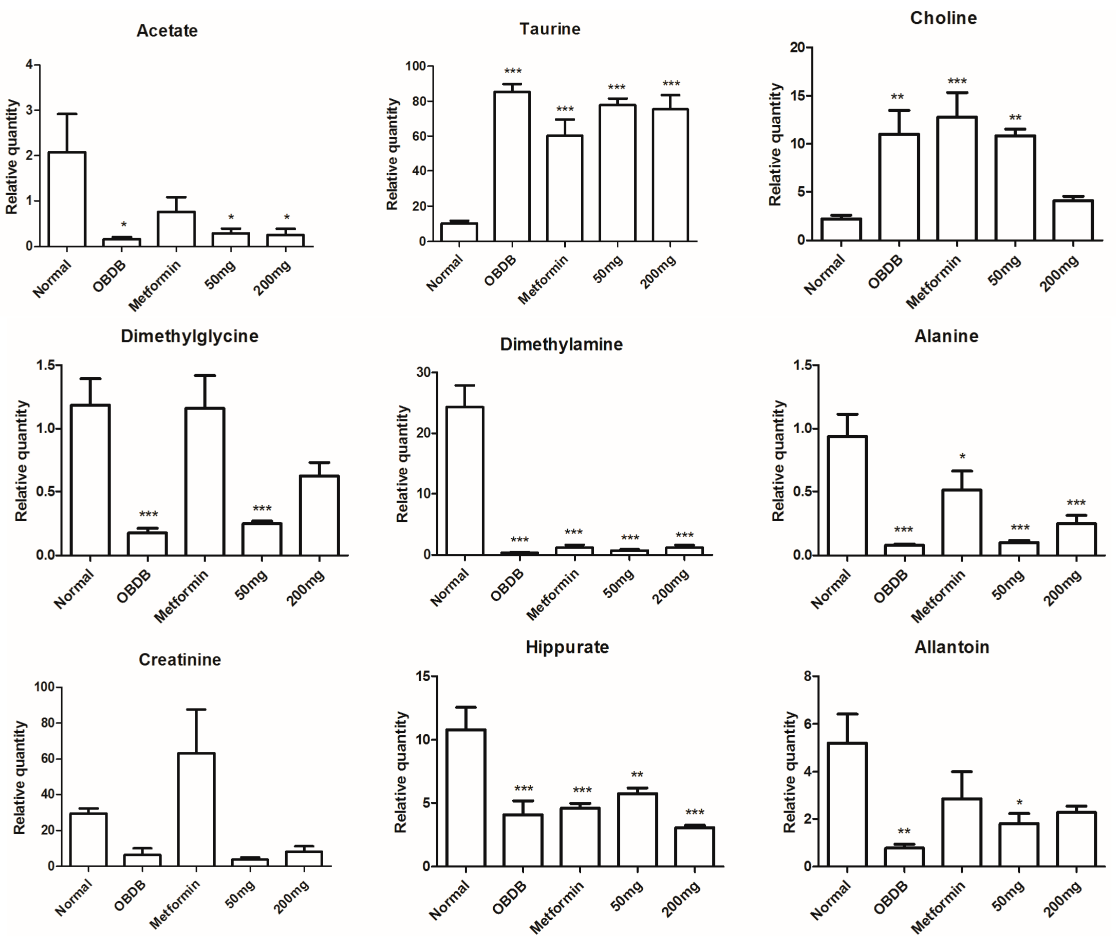

Figure S2. Relative quantification of the differentiating metabolites in urine samples (urine samples collected at basal stage) of normal, obese-diabetic (obdb), obese-diabetic rats treated with 50, 200 mg of *A. paniculata* extract and metformin. Relative quantification is based on mean peak area of the related ^{1}H NMR signals. * depict the differences between normal (control) and obese-diabetic (obdb), obese-diabetic rats treated with the different concentrations of *A. paniculata* extract and metformin. Statistical icons: * $p < 0.05$, ** $p < 0.01$ and *** $p < 0.001$.

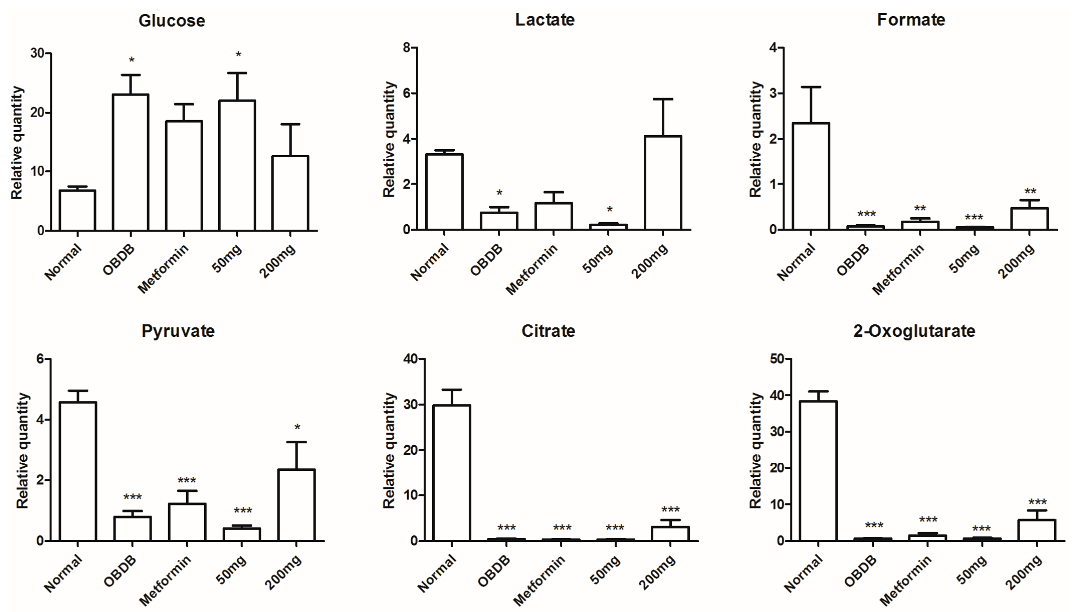

Figure S3. *Cont.*

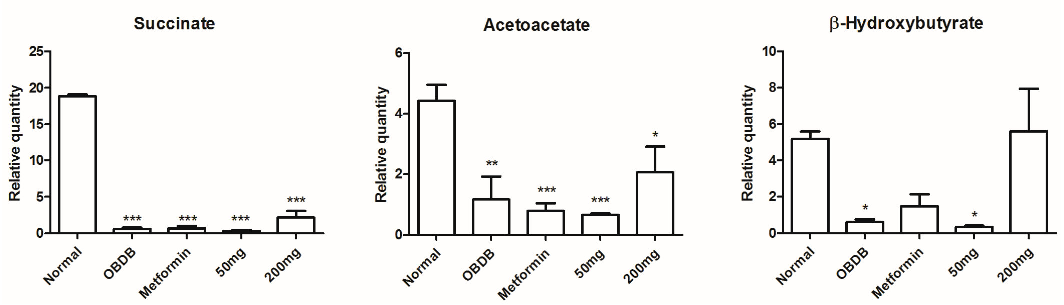

Figure S3. Relative quantification of the differentiating metabolites in urine samples (urine samples collected at middle stage) of normal, obese-diabetic (obdb), obese-diabetic rats treated with 50, 200 mg of *A. paniculata* extract and metformin. Relative quantification is based on mean peak area of the related ^{1}H NMR signals. * depict the differences between normal (control) and obese-diabetic (obdb), obese-diabetic rats treated with the different concentrations of *A. paniculata* extract and metformin. Statistical icons: * $p < 0.05$, ** $p < 0.01$ and *** $p < 0.001$.

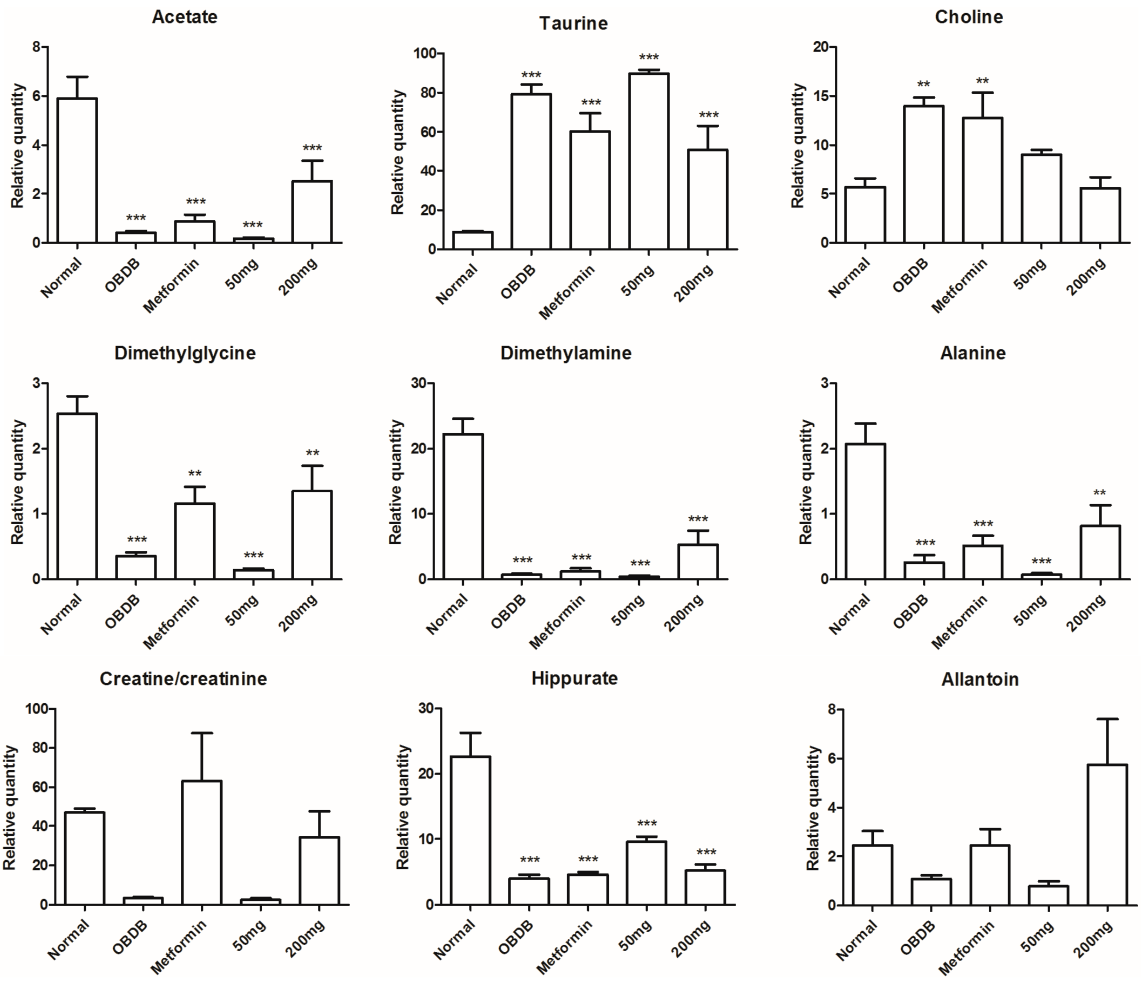

Figure S4. Relative quantification of the differentiating metabolites in urine samples (urine samples collected at middle stage) of normal, obese-diabetic (obdb), obese-diabetic rats treated with 50, 200 mg of *A. paniculata* extract and metformin. Relative quantification is based on mean peak area of the related ^{1}H NMR signals. * depict the differences between normal (control) and obese-diabetic (obdb), obese-diabetic rats treated with the different concentrations of *A. paniculata* extract and metformin. Statistical icons: * $p < 0.05$, ** $p < 0.01$ and *** $p < 0.001$.

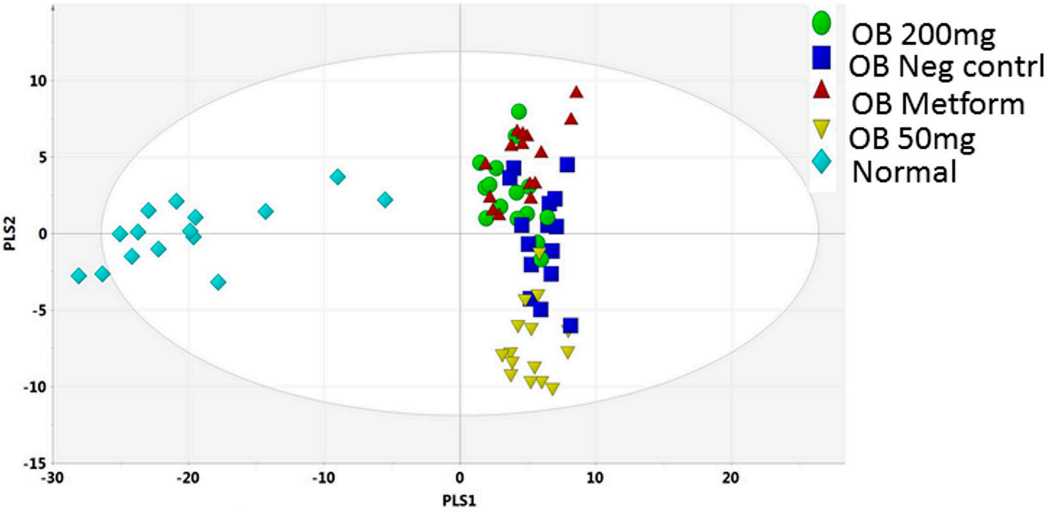

Figure S5. PCA score plot of normal, obese and obese treated (50, 200 mg of *A. paniculata* extract and metformin) rats urine samples collected at basal, middle and final stage. Normal (normal), obese (OB Neg contrl), obese rats treated with 50 mg of *A. paniculata* extract (OB 50mg), obese rats treated with 200 mg of *A. paniculata* extract (OB 200mg), obese rats treated with metformin (OB metform).

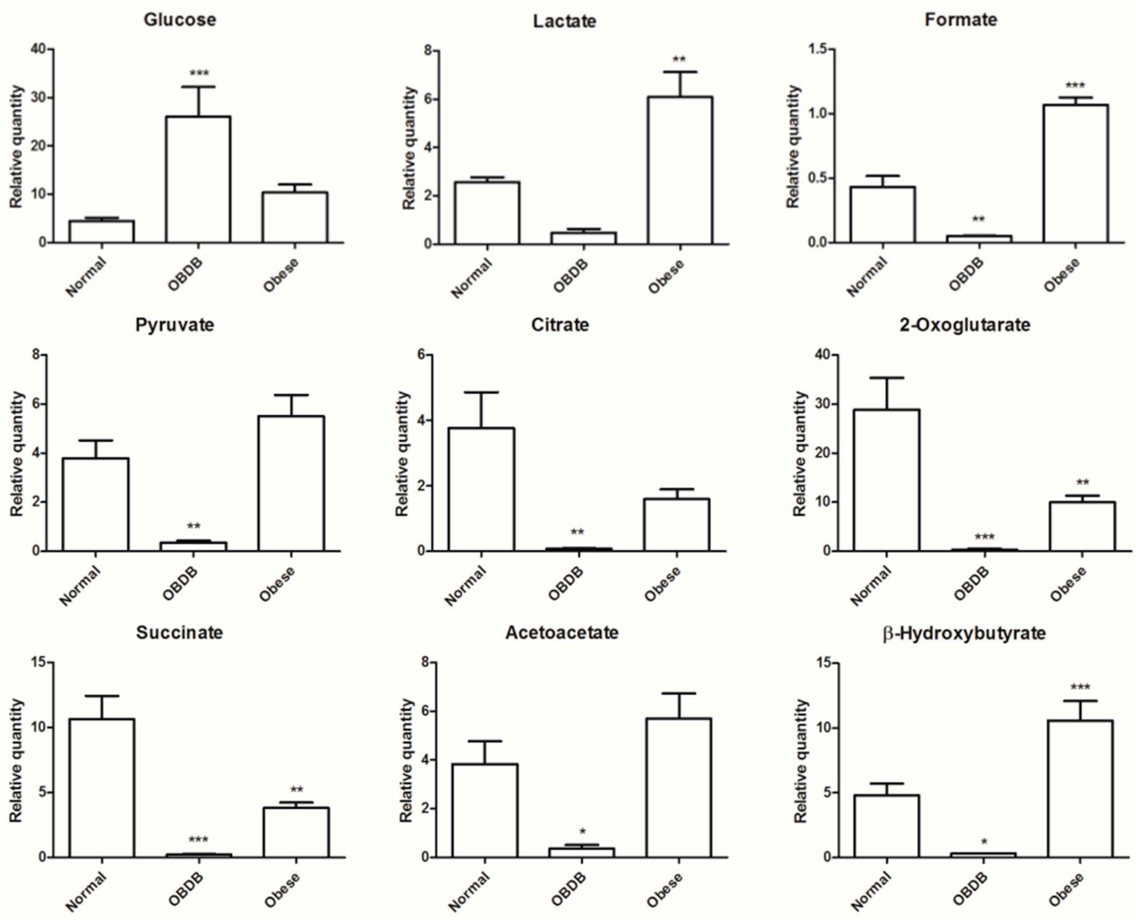

Figure S6. Relative quantification of the differentiating metabolites in urine samples (urine samples collected at basal stage) of normal, obese and obese-diabetic (obdb) rats. Relative quantification is based on mean peak area of the related ^{1}H NMR signals. * depict the differences between normal (control) and obese and obese-diabetic (obdb) rats. Statistical icons: * $p < 0.05$, ** $p < 0.01$ and *** $p < 0.001$.

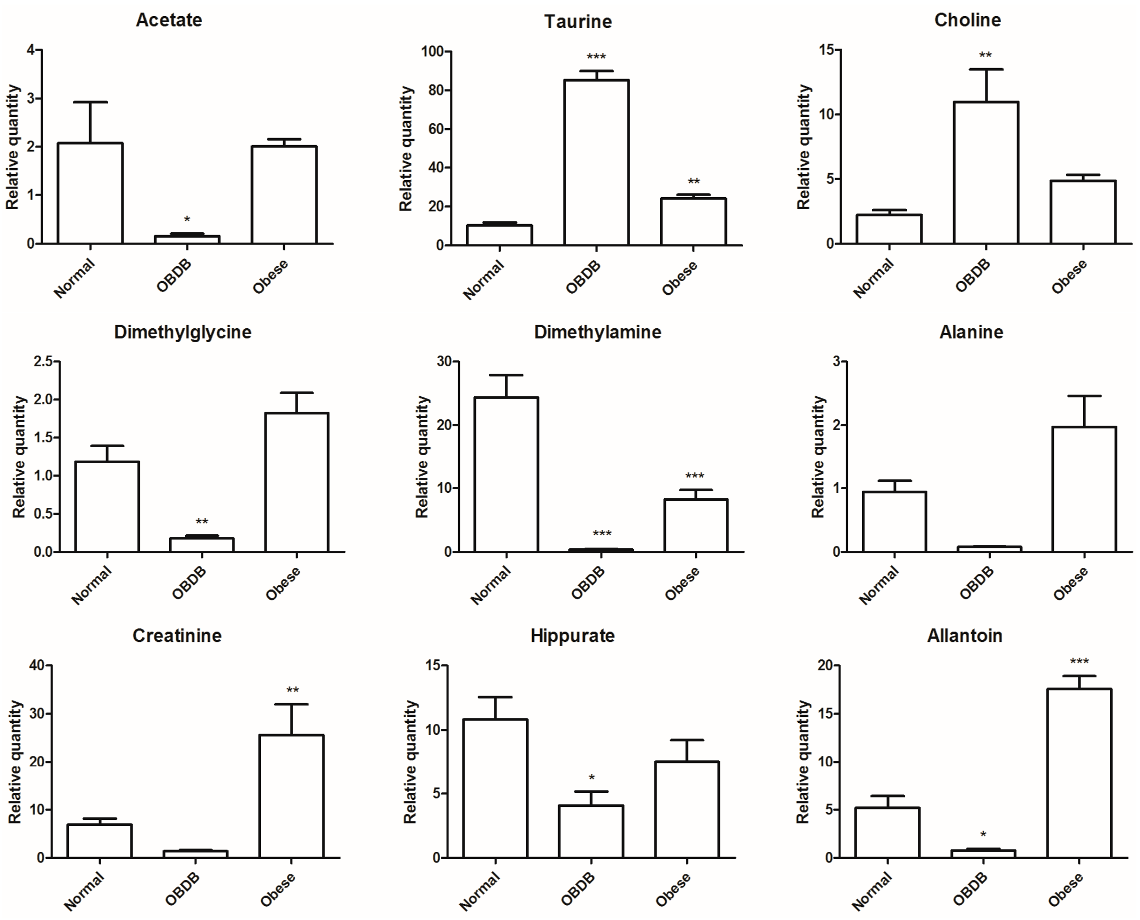

Figure S7. Relative quantification of the differentiating metabolites in urine samples (urine samples collected at basal stage) of normal, obese and obese-diabetic (obdb) rats. Relative quantification is based on mean peak area of the related ^{1}H NMR signals. * depict the differences between normal (control) and obese and obese-diabetic (obdb) rats. Statistical icons: * $p < 0.05$, ** $p < 0.01$ and *** $p < 0.001$.

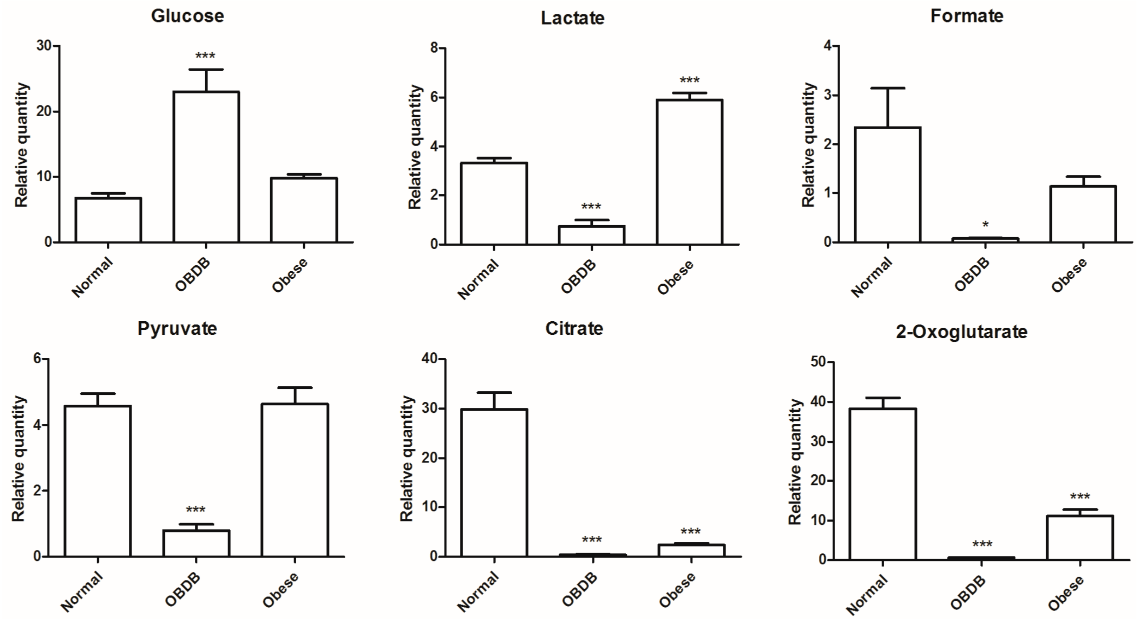

Figure S8. *Cont.*

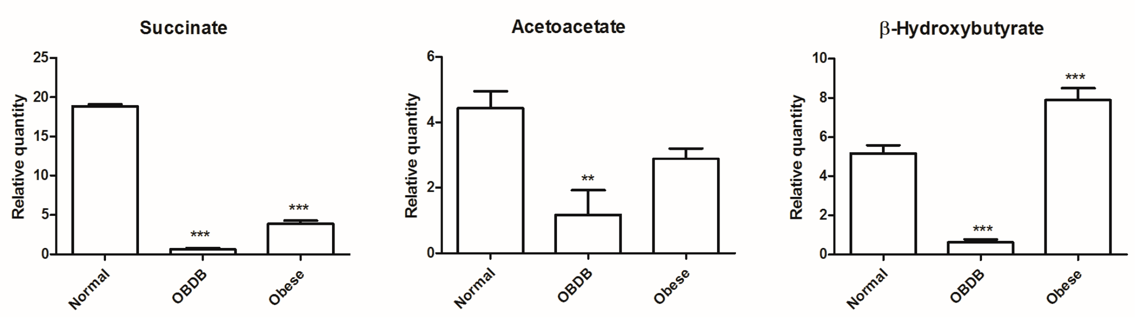

Figure S8. Relative quantification of the differentiating metabolites in urine samples (urine samples collected at middle stage) of normal, obese and obese-diabetic (obdb) rats. Relative quantification is based on mean peak area of the related ^{1}H NMR signals. * depict the differences between normal (control) and obese and obese-diabetic (obdb) rats. Statistical icons: * $p < 0.05$, ** $p < 0.01$ and *** $p < 0.001$.

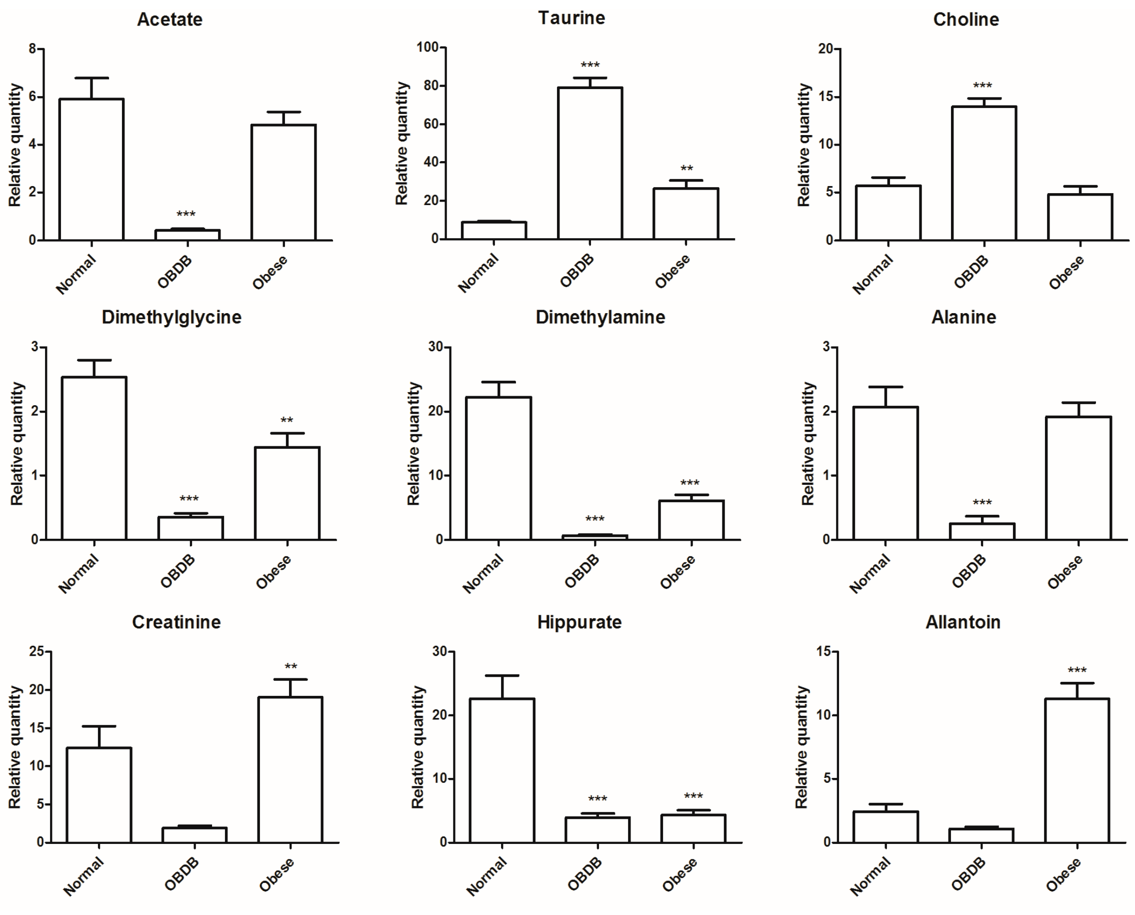

Figure S9. Relative quantification of the differentiating metabolites in urine samples (urine samples collected at basal stage) of normal, obese and obese-diabetic (obdb) rats. Relative quantification is based on mean peak area of the related ^{1}H NMR signals. * depict the differences between normal (control) and obese and obese-diabetic (obdb) rats. Statistical icons: * $p < 0.05$, ** $p < 0.01$ and *** $p < 0.001$.

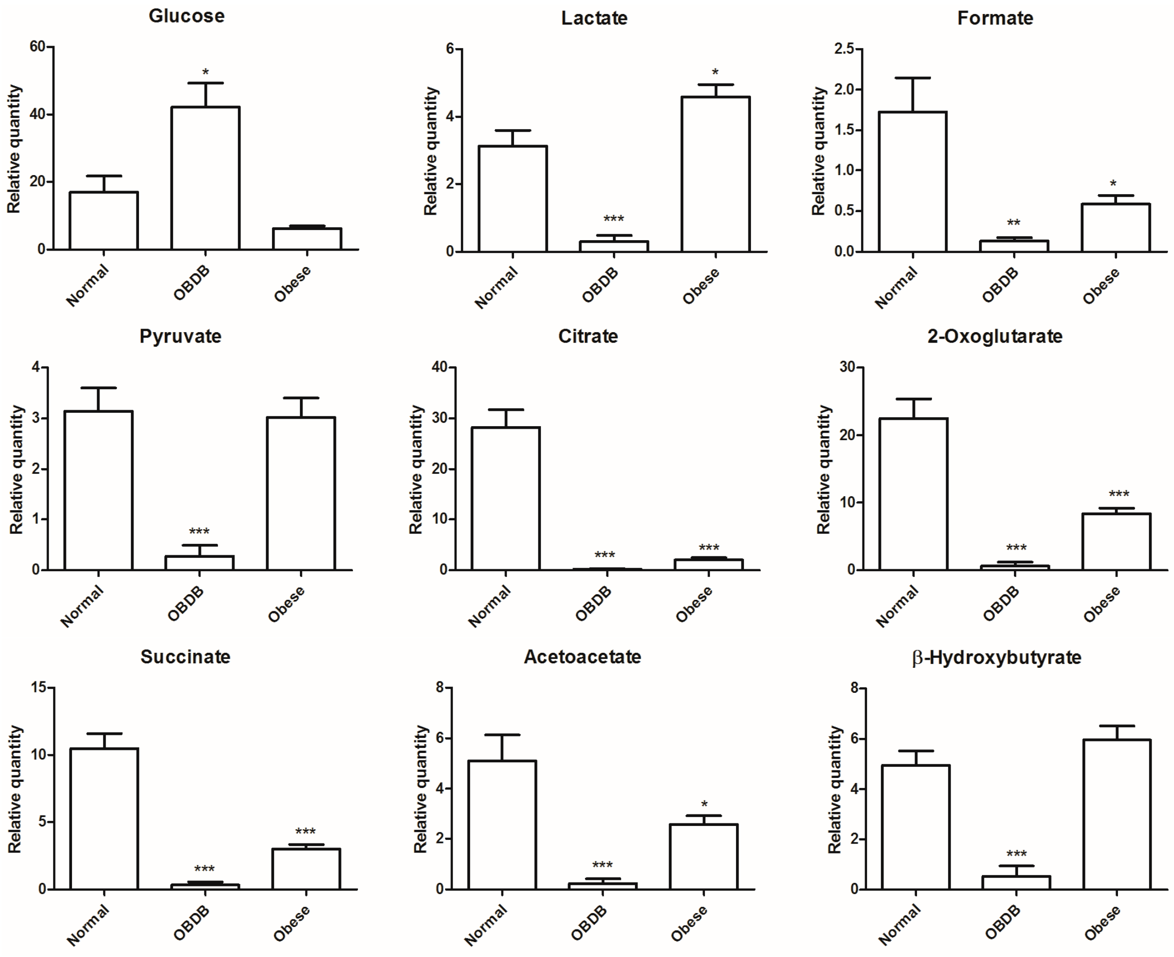

Figure S10. Relative quantification of the differentiating metabolites in urine samples (urine samples collected at final stage) of normal, obese and obese-diabetic (obdb) rats. Relative quantification is based on mean peak area of the related ^{1}H NMR signals. * depict the differences between normal (control) and obese and obese-diabetic (obdb) rats. Statistical icons: * $p < 0.05$, ** $p < 0.01$ and *** $p < 0.001$.

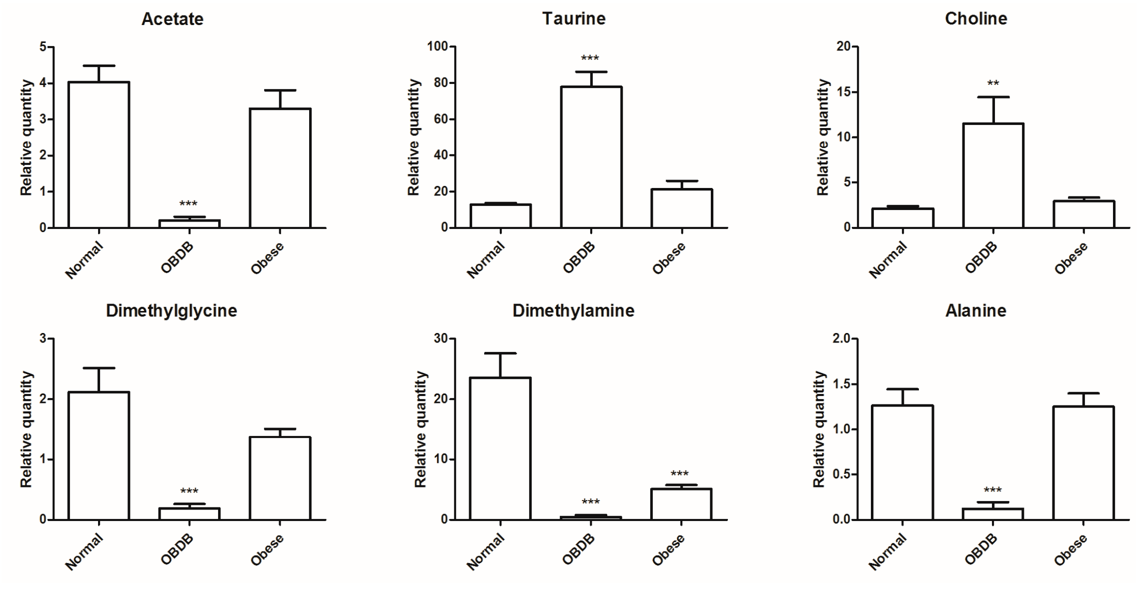

Figure S11. *Cont.*

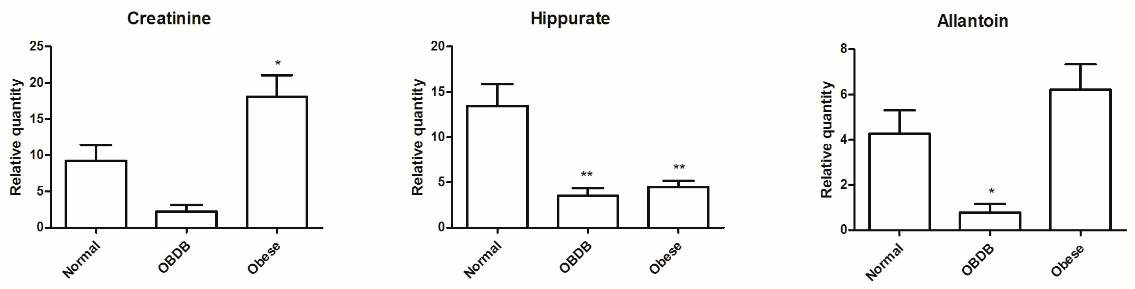

Figure S11. Relative quantification of the differentiating metabolites in urine samples (urine samples collected at final stage) of normal, obese and obese-diabetic (obdb) rats. Relative quantification is based on mean peak area of the related ^1H NMR signals. * depict the differences between normal (control) and obese and obese-diabetic (obdb) rats. Statistical icons: * $p < 0.05$, ** $p < 0.01$ and *** $p < 0.001$.
